# A case of ectopic ACTH syndrome treated with intermittent administration of dopamine agonists

**DOI:** 10.1530/EDM-14-0001

**Published:** 2014-03-01

**Authors:** Satoru Sakihara, Kazunori Kageyama, Satoshi Yamagata, Ken Terui, Makoto Daimon, Toshihiro Suda

**Affiliations:** Department of Endocrinology and MetabolismHirosaki University Graduate School of Medicine5 Zaifu-cho, Hirosaki, Aomori, 036-8562Japan

## Abstract

ACTH-dependent Cushing's syndrome includes Cushing's disease and ectopic ACTH syndrome (EAS). The differential diagnosis of Cushing's disease from EAS in cases of ACTH-dependent Cushing's syndrome is a challenging problem. We report here a case of EAS with an unknown source of ACTH secretion. Extensive imaging procedures, involving computed tomography (neck to pelvis), pituitary magnetic resonance imaging, and whole-body ^18^F-fluorodeoxyglucose-positron emission tomography, failed to reveal the source of ACTH secretion. Intermittent administration of bromocriptine, a short-acting and nonselective dopamine agonist, has afforded adequate suppression of plasma ACTH and cortisol levels over the long term.

**Learning points:**

- Tumor excision is the primary treatment for EAS. However, when surgery is impossible, medical therapy is needed to treat hypercortisolism.
- In cases where the source of ACTH secretion is unknown, inhibitors of steroidogenesis, such as metyrapone, mitotane, ketoconazole, and etomidate, are mostly used to suppress cortisol secretion.
- Medications that suppress ACTH secretion are less effective, therefore less popular, as standard treatments.
- In the present case, short-term treatment with dopamine agonists was effective for the long-term suppression of both ACTH and cortisol levels.

## Background

Cushing's syndrome is characterized by signs and symptoms resulting from chronic glucocorticoid excess. Adrenocorticotropic hormone (ACTH)-dependent Cushing's syndrome includes Cushing's disease and ectopic ACTH syndrome (EAS), with the latter resulting from an extrapituitary ACTH-secreting tumor such as a small-cell lung carcinoma, pheochromocytoma, or bronchial or thymic carcinoid (1). The differential diagnosis of Cushing's disease from EAS in cases of ACTH-dependent Cushing's syndrome is a challenging problem in clinical endocrinology because the findings for Cushing's disease partially overlap with those of EAS (2). In addition, localization of the tumor in EAS often complicates its surgical removal, which is the first-line treatment for EAS. In cases where the source of ACTH secretion is unknown, inhibitors of steroidogenesis, such as metyrapone, mitotane, ketoconazole, and etomidate, are mostly used to suppress cortisol secretion (3).

We present the case of EAS, for which extensive imaging procedures failed to reveal the source of ACTH secretion but dopamine agonists provided long-lasting control of hypercortisolemia.

## Case presentation

A 65-year-old Japanese woman was referred for evaluation of hypokalemia. On admission, her body weight was 57.0 kg and BMI was 27.5 kg/m^2^. She had no regular alcohol intake. She had no symptoms such as lethargy and change in mood. She had been diagnosed with type 2 diabetes mellitus and hypertension 1 year before, and controlled with diet and an antihypertensive agent respectively. She was still hypertensive (171/85 mmHg) and had a moon face, central obesity, buffalo hump, purple striae, thin skin, and proximal myopathy.

## Investigation

**Figure 1:**
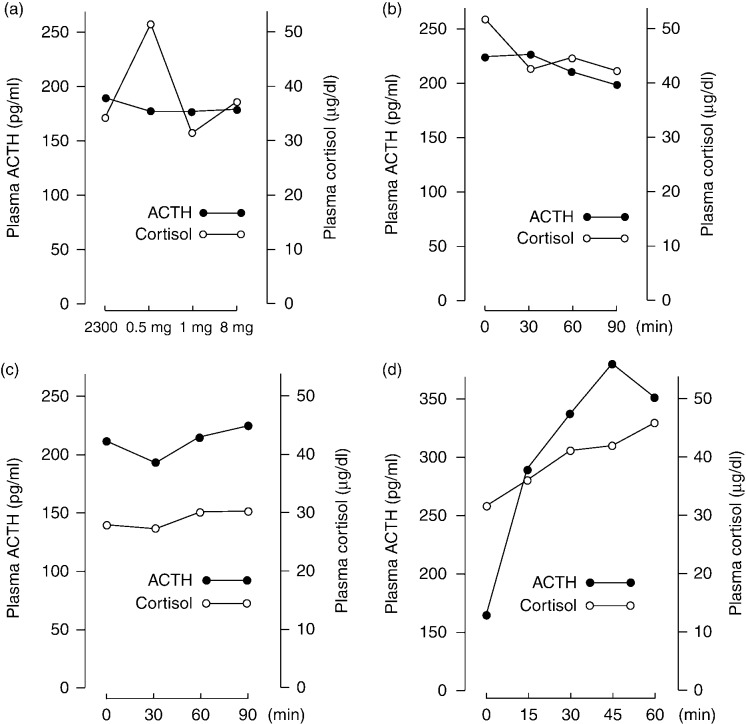
Hormone loading test results (on first administration). (a) Overnight dexamethasone suppression test (per os at 2300 h); (b) desmopressin test (4 μg, i.v. bolus); (c) human corticotropin-releasing hormone test (100 μg, i.v. bolus); and (d) growth hormone-releasing peptide 2 test (100 μg, i.v. bolus). ACTH, adrenocorticotropic hormone.

**Figure 2:**
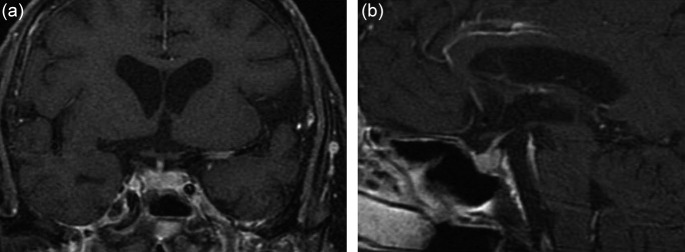
Coronal (a) and sagittal (b) MRI scans with Gd-DTPA enhancement of the pituitary.

**Figure 3:**
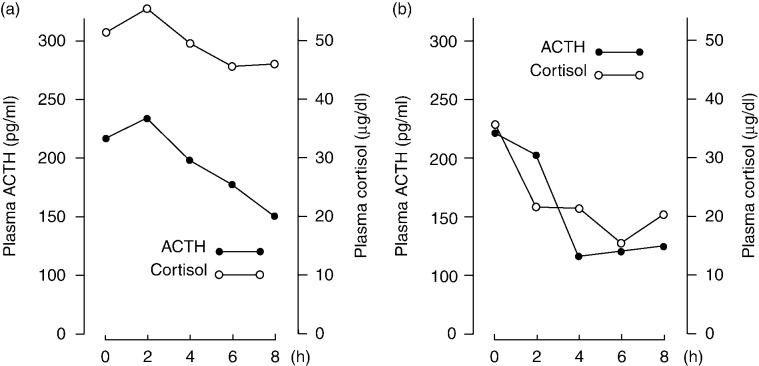
Changes in plasma adrenocorticotropic hormone (ACTH) and cortisol levels following treatment with (a) octreotide (100 μg, s.c.) or (b) bromocriptine (2.5 mg, per os in the morning) alone (first administration).

All endocrine examinations were performed according to clinical guidelines, and the patient provided informed consent for the tests. The plasma basal ACTH level was 225 pg/ml (49.6 pmol/l) and the plasma cortisol level was 54.8 μg/dl (1512.5 nmol/l). Excretion of urinary free cortisol was 1099 μg/day (3033.2 nmol/day). As the laboratory data suggested ACTH-dependent hypercortisolism, further tests were performed to determine autonomic secretion of ACTH. They revealed i) incomplete suppression of plasma cortisol levels (>5 μg/dl (133.5 nmol/l)) in a low-dose (0.5 mg) overnight dexamethasone suppression test (DST), ii) high plasma cortisol levels (>5 μg/dl (133.5 nmol/l)) during nighttime sleep, and iii) no response of plasma ACTH levels to the desmopressin (DDAVP) test (Fig. 1). Additional endocrine examinations demonstrated the following: no response of plasma ACTH levels by the human corticotropin-releasing hormone (hCRH) test; an exaggerated response of plasma ACTH levels by the growth hormone-releasing peptide 2 (GHRP2) test; little suppression of plasma cortisol levels (less than half the basal level) in a high-dose (8 mg) overnight DST; and no clear pituitary adenoma and no shift of pituitary stalk on MRI with Gd-DTPA enhancement (Fig. 2). A selective venous sampling test was also performed, and catheterization of only the right side was successful. As plasma ACTH levels increased after the GHRP2 test, but not after the hCRH test, sampling was followed by the administration of GHRP2. The results of cavernous sinus sampling were considered diagnostic for an ectopic source of ACTH production because the basal ratio (central:peripheral ratio before the test) was 1.16, lower than the expected 2.0, and the peak ratio after GHRP2 administration was 1.05 (Table 1). However, CT scans from neck to pelvis failed to detect localization of an ectopic source, as did FDG-PET. Both plasma ACTH and cortisol levels were weakly decreased by the test dose of somatostatin analog octreotide and potently suppressed by the dopamine agonist bromocriptine (Fig. 3).

**Table 1:** Cavernous sinus sampling

	**ACTH** (pg/ml)	
	Basal	After GHRP2
Inferior vena cava	174	348
Right jugular vein	184	389
Right inferior petrosal sinus	181	370
Right cavernous sinus	201	366

ACTH, adrenocorticotropic hormone; GHRP2, GH-releasing peptide.

## Treatment

**Figure 4:**
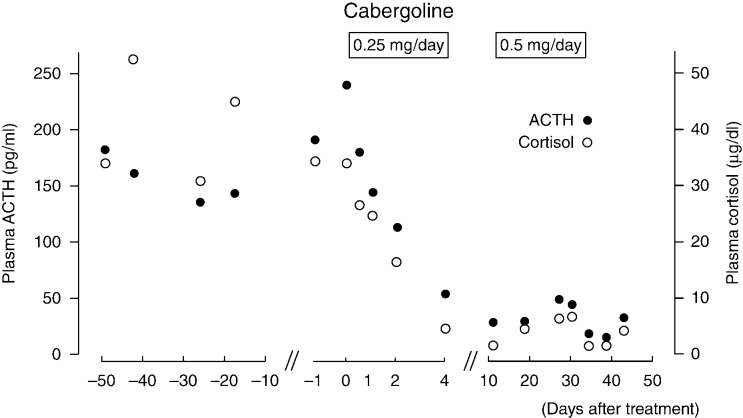
Clinical course of changes in plasma ACTH and cortisol levels before and after cabergoline treatment (2 years later).

Based on a response to the test dose of bromocriptine, the patient was treated with selective dopamine agonist cabergoline at 0.25 mg/day (Fig. 4), which was gradually increased to 0.5 mg/day and maintained plasma ACTH and cortisol levels, at lower normal range for 40 days (Fig. 4).

## Outcome and follow-up

**Figure 5:**
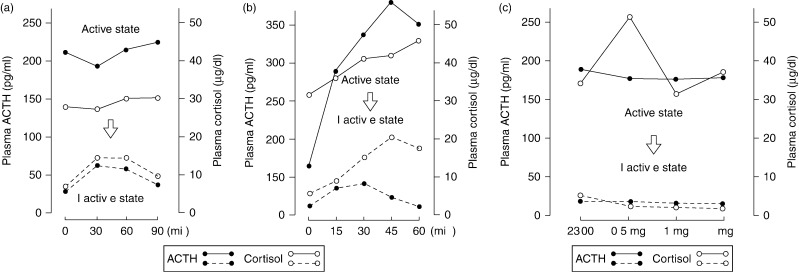
Hormone loading test results during active and inactive states. (a) Human corticotropin-releasing hormone test (100 μg, i.v. bolus); (b) growth hormone-releasing peptide 2 test (100 μg, i.v. bolus); and (c) overnight dexamethasone suppression test (per os at 2300 h). ACTH, adrenocorticotropic hormone.

Cabergoline was discontinued due to adrenal failure. Both basal plasma ACTH and cortisol levels stayed within normal limits and showed normal responses to hormonal tests for more than 1 year (Fig. 5). However, 16 months later, high glucose, ACTH, and cortisol levels were again observed. FDG-PET revealed a lesion in the right lobe of the thyroid. It was resected and histologically diagnosed as papillary carcinoma. However, it showed no ACTH immunostaining. Short-term treatment with cabergoline normalized plasma ACTH and cortisol levels for 1 year, but thereafter glucose, ACTH, and cortisol levels were once again increased. GH and prolactin levels were 0.11 and 5.2 ng/ml respectively. Fasting blood glucose, LDL cholesterol, and triglyceride levels were 156, 97, and 95 mg/dl respectively.

**Figure 6:**
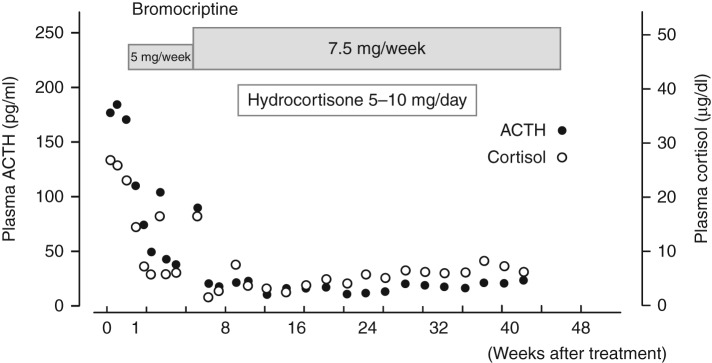
Clinical course of changes in plasma ACTH and cortisol levels following bromocriptine treatment.

As we considered that a short-acting dopamine agonist might be enough to control cortisol levels, the patient was initially treated with 2.5 mg bromocriptine two times per week, followed by 2.5 mg bromocriptine three times per week, together with replacement of hydrocortisone (5–10 mg/day). Thereafter, plasma ACTH and cortisol levels have been controlled within normal limits for almost 1 year (Fig. 6). After treatment, fasting blood glucose, LDL cholesterol, and triglyceride levels were 86, 75, and 105 mg/dl respectively.

## Discussion

The findings for Cushing's disease partially overlap with those in some cases of EAS. A low-dose DST is indispensable in screening for Cushing's syndrome, and 0.5 mg dexamethasone is used in this test in Japan (2). After correcting s.d. to within 10%, our previous data revealed that the 5.0 μg/dl cut-off had 100% sensitivity and 98% specificity for the diagnosis of Cushing's disease (4) and 100% of both for the diagnosis of EAS (5). In response to DDAVP, plasma ACTH levels were found to be significantly increased in 86% (19/22) of cases with Cushing's disease, especially those with microadenoma (90%), and in 44% (4/9) of EAS cases (5). Other studies have shown that 30–60% of EAS cases responded to DDAVP. These results suggest that the DDAVP test cannot be used to effectively discriminate Cushing's disease from EAS.

In our previous study, plasma ACTH levels were increased by more than 1.5-fold in response to hCRH in almost all cases of Cushing's disease, including 100% (62/62) and 73% (8/11) in cases with microadenoma and macroadenoma respectively (5). On the other hand, 27% (4/15) of EAS cases also responded to hCRH (5). Although the hCRH test may be effective for distinguishing Cushing's diseases from EAS, definitive discrimination between them using this test alone is difficult. Our previous results showed that a high-dose (8 mg) DST for diagnosing Cushing's disease was efficacious in almost 80% of cases (5). In cases of EAS, high-dose dexamethasone suppressed three of the six bronchial carcinoids and none of the nine other lung cancers (5). These results suggest that a high-dose dexamethasone test is useful for diagnosing Cushing's disease, although caution is required when interpreting the results in cases of macroadenoma or Crooke cell adenoma with Cushing's disease and in cases of bronchial carcinoid with EAS. When applying both the hCRH test and 8-mg DST together, our previous study revealed 81% sensitivity and 60% specificity in cases of Cushing's disease (5). Isidori *et al*. (6) reported that a low-dose DST, combined with the CRH test, obtained a higher sensitivity and specificity. As the combination of these two tests is effective for distinguishing Cushing's diseases from EAS, the data in the present case are consistent with a case of EAS.

GHRPs may have potential for screening ACTH-dependent Cushing's syndrome as plasma ACTH levels are greatly increased in response to GHRP administration in patients with Cushing's disease compared with normal subjects. Furthermore, GHRP2 is currently approved for clinical use in Japan. However, the mechanisms underlying the ACTH response in ACTH-dependent Cushing's syndrome remain unclear. MRI has shown high specificity in the diagnosis of Cushing's disease, and when combined with both the hCRH test and 8-mg DST, diagnostic specificity is even higher, at 90% in the presence of a pituitary adenoma. The ability of MRI to detect pituitary ACTH-secreting adenoma in patients with Cushing's disease is limited because the calculated accuracy for detecting a pituitary source of ACTH is reportedly around 60% with conventional MRI. However, the ability to detect pituitary ACTH-secreting adenoma might be improved by using a 3 Tesla magnet, which is also suitable for detecting small nonfunctioning adenoma. Cavernous or inferior petrosal sinus sampling has been validated as a highly accurate investigative tool for the differential diagnosis of ACTH-dependent Cushing's syndrome. Administration of hCRH to stimulate ACTH secretion during sampling is routinely used to elicit diagnostic gradients in cases of Cushing's disease and to improve the sensitivity of this procedure. Gradients of sinus (central) to peripheral ACTH are calculated before and after stimulation with hCRH, and Cushing's disease is diagnosed when the basal gradient is >2 or when the gradient after the stimulation is >3. The basal ratio of cavernous sinus sampling in our case was considered diagnostic for an ectopic/peripheral source of ACTH production. However, a unilateral petrosal sinus sampling failed to exclude Cushing's disease perfectly, because catheterization of the left side was unsuccessful. In our case, central ACTH levels failed to be increased after the stimulation. GHRP2 stimulation would not be interpreted, because there are no data available on its use in differential diagnosis of ACTH-dependent Cushing's syndrome. The endocrine findings in our case are mostly consistent with an ectopic ACTH-secreting tumor. EAS is most frequently caused (in ∼50% of all cases) by a bronchial carcinoid or small-cell lung carcinoma. However, a thymic carcinoid, gastroenteropancreatic neuroendocrine tumor, pheochromocytoma, or medullary thyroid carcinoma may also cause this syndrome. In most cases, the tumor is identified by routine imaging procedures; CT is a useful modality for localizing an ectopic source and, in some cases of EAS, PET scans are helpful for detecting localization. In addition, somatostatin receptor imaging, which is not available commercially in Japan, complements radiological imaging in localizing ectopic ACTH secretion sites, while glucocorticoid-lowering or -antagonizing therapy has the potential to improve the diagnostic accuracy of somatostatin receptor scintigraphy.

Despite extensive diagnostic procedures, the source of ACTH secretion remained occult in our case. Tumor excision is the primary treatment for EAS. However, when surgery is impossible, medical therapy is needed to treat hypercortisolism. The spectrum of available drugs includes adrenal-blocking agents, neuromodulatory drugs, and glucocorticoid receptor antagonists (7). Adrenal-blocking drugs suppress adrenal cortisol production by inhibiting steroidogenic enzymes (7), and prolonged remission after long-term treatment with steroidogenesis inhibitors in Cushing's syndrome has been reported in cases of EAS (8). Drugs that suppress ACTH secretion are less popular as standard treatments and may include cyproheptadine, valproic acid, cabergoline, somatostatin analogs, peroxisome proliferator-activated receptor γ agonists, and vasopressin antagonists (9).

The first studies of dopamine agonists in Cushing's disease were performed with bromocriptine; however, the effect was not strong and was maintained over the longer term in only a small subset of cases. Better results were expected with cabergoline, which has a higher binding capacity to the dopamine type 2 receptor and a longer half-life. Pivonello *et al*. (10) reported six cases of ACTH-secreting carcinoids in patients who had undergone surgery, where dopamine receptors were expressed in neuroendocrine tumors associated with EAS, and cabergoline treatment was effective for controlling the cortisol excess in the subgroup of patients with EAS (10). Expression of the short isoform of the dopamine type 2 receptor, and/or coexpression of the dopamine type 4 receptor, may play a pivotal role in the effectiveness of dopamine agonists in carcinoid tumors associated with EAS (11). In the present case, short-term initial treatment with cabergoline was effective for the long-term suppression of both ACTH and cortisol levels. Spontaneous remission in Cushing's syndrome is rare (8), and the possibility of cyclic ACTH production cannot be completely excluded. Finally, intermittent administration (three times a week) with bromocriptine, a short-acting (*T*_1/2_=2.86 h) dopamine agonist, has afforded adequate suppression of ACTH levels over the long term. Therefore, it is possible that treatment with the dopamine agonist may have deactivated ACTH secretion in this case.

The case still might have a possibility of a pituitary dysregulation of ACTH secretion that was sensitive to dopamine agonists, because the findings for Cushing's disease partially overlap with those in some cases of EAS. Although dopamine agonists deactivated ACTH secretion, it does not mean that they have also killed the occult tumor. The patient needs to be followed up by extensive examinations, including laboratory tests and imaging procedures.

In summary, a case of EAS with an unknown source of ACTH secretion was successfully controlled by administering the short-acting dopamine agonist bromocriptine.

## Patient consent

Written informed consent has been obtained from the patient for publication of this case report.

## Author contribution statement

S Sakihara is the main Endocrinologist Physician who followed the patient. K Kageyama is also an Endocrinologist Physician and responsible for case description. S Yamagata and K Terui are Endocrinologist Physicians following the patient at present. M Daimon is the present director of the Department of Endocrinology and Metabolism. T Suda is the previous director of the Department of Endocrinology and Metabolism.
